# Efficacy of Half-Day Workshops for Internal Medicine Interns in Educating Breaking-Bad-News Discussions

**DOI:** 10.1089/pmr.2020.0097

**Published:** 2021-05-17

**Authors:** Colin Thomas, Christine Kurian, Sarah Houtmann, Neil Palmisiano

**Affiliations:** ^1^ Department of Internal Medicine, Thomas Jefferson University Hospital, Philadelphia, Pennsylvania, USA.; ^2^ Department of Medical Oncology, Sidney Kimmel Cancer Center at Thomas Jefferson University Hospital, Philadelphia, Pennsylvania, USA.

**Keywords:** breaking bad news, end-of-life care, resident education

## Abstract

***Background:*** Adequate end-of-life (EOL) care/breaking-bad-news (BBN) discussions with patients are becoming increasingly essential to adequate patient care.

***Purpose:*** Whether a half-day workshop would lead to improved confidence in EOL/BBN care discussions for internal medicine interns.

***Methods:*** Internal medicine interns (*n* = 43) were assigned to participate in a half-day workshop at Thomas Jefferson University Hospital. The workshop involved two standardized patient (SP) interactions involving delivering news of a terminal illness/initiating goals of care discussion with the intervention of SP feedback, a didactic and lecture on proper EOL/BBN discussion. Voluntary anonymous surveys before and after the workshop were utilized to assess impact.

***Results:*** A majority of interns felt more comfortable with leading EOL care/BBN discussions after the workshop and had a positive experience.

***Conclusions:*** A half-day curriculum is efficacious in educating EOL/BBN communication to internal medicine interns, but should be further assessed in a larger more standardized study involving an objective assessment.

## Introduction

Preparing postgraduate year-1 (PGY-1) internal medicine interns for emotionally difficult end-of-life (EOL)/breaking-bad-news (BBN) discussions with patients and their families is not only essential to training skilled physicians, but also in providing quality care to the patients of the residency's associated academic hospital.^1,2^ EOL care/BBN has also been at the forefront of discussion in the context of the U.S. health care system. Recent data suggest that one in five deaths in the United States involve intensive care services—significantly more than most Western countries, without the benefit of improved mortality; of note, when polled, most Americans, >90%, do not wish to die in the hospital, but rather at home.^1^

In addition, in some cases it has been determined that early palliative intervention for patients with terminal diseases improves both quality of life and survival, thereby stressing the importance of effective EOL discussions.^2^ Early EOL discussions have been shown to be associated with less aggressive care and more use of hospice^3^; it has also been shown that many patients diagnosed with terminal diseases, such as metastatic malignancies, had not had EOL care options discussed after diagnosis.^4^

Although residents seem to become more comfortable with EOL/BBN discussions with more exposure throughout training, many residents continue to feel uncomfortable and conflicted having EOL/BBN care discussions with their patients.^5^ One study found that internal medicine residents can have an average of approximately six EOL/BBN discussions per month, whereas receiving little feedback on their skills from their residency program during their training.^6^

There have been several published studies in determining effective training methods for residents in EOL/BBN discussion; one literature review assessed 21 different studies on the subject with various methods of intervention—including didactics, designated rounds, simulations, and group discussions; most of the studies reported improvement in knowledge, attitudes, and skills involved with EOL issues.^7^ One study evaluated whether intervention through online videos of EOL discussion education would be effective in educating residents; however, their data did not show a significant improvement in EOL discussions skills, highlighting the importance of face-to-face interaction in teaching these skills.^8^

With regard to in-person training sessions, a study at Duke University Medical Center evaluated small-group teaching workshops through a two-day retreat composed of 16 hours of educating pain/symptom management and communication skills; their study noted an improvement in EOL/BBN communication skills among their residents through a scoring system graded during standardized patient (SP) interactions before and after their intervention.^9^ A later study out of Brigham and Women's Hospital (BWH) assessed the efficacy of a similar workshop conducted over the course of a day, utilizing five hours of teaching with assessment through SP interaction before and after the intervention; their study found significant improvement in EOL conversation performance.^10^

Given the success of both the BWH and Duke studies, we are interested in evaluating a similar design, but in a more condensed time frame; ultimately, utilizing a workshop involving ∼1.5 hours of lectures and small-group discussion as compared with 5 and 16 hours of teaching in the BWH and Duke studies, respectively. Residents, most notably PGY-1s, have busy schedules and an effective training session that can more easily fit their schedule would be of great value to a residency program.

In addition, the BWH and Duke studies evaluated only PGY-2s exclusively or combination of PGY-1s, 2s, and 3s, respectively. Our study is aimed at targeting the workshop intervention to only PGY-1 internal medicine interns, since we feel that it is of great importance to start this intervention early in internal medicine residency: thereby determining, by our study, if such small-group workshops would be efficacious for groups comprised solely of PGY-1s. Focusing on internal medicine interns is especially important as EOL care/BBN discussion skills are mentioned as a learning requirement in the Accreditation Counsel for Graduate Medical Education (ACGME) guideline for Internal Medicine Residency programs.^11^

## Methods

Anonymous surveys of staff at Thomas Jefferson University Hospital are categorized as “minimal to no risk” and do not require Institutional Review Board (IRB) review. A model schedule for the half-day workshop is shown in Table 1 and the cases utilized by the SP interactions #1 and #2 are outlined in Table 2 as Case #1 and Case #2, respectively. Internal medicine interns, part of the Thomas Jefferson University Hospital's internal medicine residency, were asked to volunteer in this study. A total of 43 interns, including 36 categorical internal medicine interns and 7 noncategorical interns, participated in the half-day workshop, which encompassed all of the internal medicine interns at our academic institution. The workshop took place during protected time during ambulatory/elective rotations within the first half of the academic year.

**Table 1.** Sample Half-Day Workshop Schedule

Preworkshop survey to be completed before attending.
8:00 AM–9:15 AM: SP interaction #1
9:15 AM–9:40 AM: Small-group discussion: “What's difficult about having EOL conversations for you?”
9:40 AM–10:30 AM: PowerPoint lecture on modeling a conversation
10:15 AM–11:30 AM: SP interaction #2
Postworkshop survey to be completed after SP interaction #2.

A preworkshop and postworkshop survey was completed by internal medicine interns participating in the session.

EOL, end-of-life; SP, standardized patient.

**Table 2.** Cases for Standardized Patient Interactions

SP case #1:
Patient is coming to your outpatient primary care clinic to follow up the result of an outpatient CT of the abdomen/pelvis. The patient has history of hypertension and diet-controlled diabetes who has been having unintentional weight loss (5.5 kg over three months) and new painless jaundice for the past four weeks. An outpatient CT of the abdomen/pelvis was done and was read by the radiologist as concerning for incurable metastatic pancreatic cancer.
You are instructed to explain the diagnosis, poor prognosis, and begin to address goals of care.
SP case #2:
Patient was transferred from an outside hospital with new epistaxis and irregular cell counts on workup at the outside hospital emergency room. The patient is currently on your inpatient hospital service awaiting the results of the initial workup. After the flow cytometry and bone marrow biopsy result, it is evident that the patient has a new diagnosis of an aggressive poor prognosis acute myeloid leukemia. The patient is not a candidate for a curable transplant.
You are instructed to explain the diagnosis, poor prognosis, and begin to address goals of care.

Case #1 was used for the first SP interaction and case #2 was used for the second SP interaction.

The participating internal medicine interns were required to participate in the workshop as part of a training requirement in the Thomas Jefferson University Hospital internal medicine residency program; however, participation in the study (i.e., answering anonymous preworkshop and postworkshop surveys) was voluntary. The anonymous and voluntary nature of the survey was emphasized to each participating intern; the surveys did not include any identifying information about each participating trainee. Survey questions were loosely based on the survey questions utilized in the Szmuilowicz et al. article.^10^ All 43 interns participated in the preworkshop survey; however, only 31 interns completed the postworkshop survey; 12 interns were lost to follow-up in the postworkshop survey. The survey questions and responses are outlined in Table 3.

**Table 3.** Intern Responses from Survey Answered Before the Workshop and the Survey Answered After the Workshop

Preworkshop survey (n = 43)	Every day	Few times per week	Once per week	Few times per month	Once per month	Less than once per month
How many times as an intern have you had to deliver bad news to a patient and/or their family?						
Number answered	0	1	4	16	13	9
Percentage	0	2.33	9.30	37.21	30.23	20.93
How many times have you had to discuss code status with a patient and/or family member?						
Number answered	1	13	14	10	4	1
Percentage	2.33	30.23	32.56	23.36	9.30	2.33
	Strongly agree	Agree	Neutral	Disagree	Strongly disagree	
I feel comfortable delivering bad news to a patient and/or family member.						
Number answered	6	16	11	10	0	
Percentage	13.95	37.21	25.58	23.36	0	
I feel comfortable leading a goals of care discussion with a patient and/or their family.						
Number answered	4	14	11	13	1	
Percentage	9.30	32.56	25.58	30.23	2.33	
Postworkshop survey (*n* = 31)						
	Strongly agree	Agree	Neutral	Disagree	Strongly disagree	
I feel more comfortable with EOL care discussions after the workshop.						
Number answered	6	23	2	0	0	
Percentage	19.35	74.19	6.45	0	0	
I found the workshop useful.						
Number answered	11	18	2	0	0	
Percentage	35.48	58.06	6.45	0	0	
I generally felt more comfortable during the second SP encounter than the first.						
Number answered	9	16	5	1	0	
Percentage	29.03	51.61	16.13	3.23	0	
I will take the skills I learned during the workshop with me in future hospital/clinical work.						
Number answered	18	11	2	0	0	
Percentage	58.06	35.48	6.45	0	0	

A total of 43 interns answered the preworkshop survey and a total of 31 interns answered the postworkshop survey.

SPs utilized in the workshop were apart of Jefferson's SP Program, which is a program dedicated to delivering high-quality SP encounters. Thomas Jefferson University SPs at the Sidney Kimmel Medical College are vigorously trained in all manner of cases for clinical examinations. A total of two SP actors/actresses were used for each half-day workshop with a total of 7–8 interns for each session. There were a total of six workshops on various days that spanned from August 2019 to October 2019, which were fit within the first half of the academic year for the internal medicine interns.

The clinical cases utilized by the SPs outlined in Table 2 were chosen to emphasize the BBN aspect of EOL care discussions. Both Case #1 and Case #2 involve telling a patient about a new diagnosis of an incurable disease with poor prognosis. For each clinical situation, the intern was asked to communicate the diagnosis, poor prognosis, and to begin addressing goals of care. The trained SPs, a part of Jefferson's SP Program, were given the case information, outlined in Table 2, the day before each workshop.

In addition to brief two-minute feedback provided by the SP after each session, the remaining intervention involved a small-group discussion and PowerPoint lecture led by a medical oncologist experienced in EOL/BBN communication. The small-group discussion was open ended with interns sharing their own experiences and why EOL/BBN care communication is difficult for them. The PowerPoint presentation examined proper EOL/BBN communication skills, especially highlighting the SPIKES protocol.^12^ Table 1 outlines the structured time of the small-group discussion and lecture in between the two SP interactions.

## Results

The results of the preworkshop and postworkshop anonymous surveys are outlined in Table 3. The preworkshop survey included four questions aiming to assess the comfort level and experience of the interns in leading EOL/BBN discussions with patients and their families. Approximately 79% of the interns (34/43) attested to having at least one interaction per month of delivering bad news to a patient and/or their family. Regarding code status discussions, about 88% of the interns (38/43) admitted to having more than one of these conversations per month with patients and/or their families. Before the workshop, ∼51% (22/43) interns agreed that they felt comfortable delivering bad news to a patient and/or their family member, whereas ∼42% (18/43) agreed to feeling comfortable leading a goals of care discussion with a patient and/or family member.

The postworkshop anonymous survey outlined in Table 3 included four questions aiming to assess the efficacy of the workshop. Thirty-one of the original 43 interns (∼72%) participated in the postworkshop survey; 12 of the interns were lost to follow-up. Approximately 93% of the interns (29/31) felt that the workshop was overall useful to them, with the same number of interns stating that they would take the skills that they learned from the workshop with them to future hospital/clinical interactions. Approximately 93% of the interns (29/31) felt more comfortable with EOL care discussions at the end of the workshop, with ∼81% of the interns (25/31) stating that they felt generally more comfortable with the second SP interaction.

## Conclusions

Given the importance of adequate EOL/BBN discussion in patient care, being able to properly educate internal medicine interns on the subject is critical. As evident by the preworkshop survey in our study, the majority of internal medicine interns in our program admitted to feeling uncomfortable with leading EOL discussions, whereas only about half of the interns stated they felt comfortable delivering bad news to patients. Interestingly, the majority of the interns surveyed admitted to discussing EOL care issues or BBN at least once per month with patients and/or their families, which is certainly an issue given their cited discomfort on the topic. Prior studies have shown efficacy in both multi- and single full-day workshops in teaching EOL/BBN skills; however, given the duration of such workshops, incorporating them into the busy schedules of internal medicine interns is not as practical.^9,10^ In this study, we demonstrate the efficacy of a half-day workshop.

The intervention of the workshop incorporated brief feedback from the SPs, a small-group discussion allowing the interns to discuss the difficulties of EOL/BBN communication and a PowerPoint lecture teaching the SPIKES protocol.^12^ As evident by our postworkshop study, the majority of our interns admitted to feeling more comfortable with EOL care/BBN communication after the workshop and generally felt more comfortable during the second SP interaction, thereby highlighting the improvement in confidence the interns experienced during the workshop.

An important issue with the study includes a variability in the SPs among the different days the workshops were held. Although each intern interacted with the same SP for the first and second SP session in the workshop, different SPs were used among the various days the workshops were held. This issue prevented a standardized scoring system to be in place, which was adopted by the multi- and single full-day workshops published in Alexander et al. and Szmuilowicz et al.^9,10^; these studies objectively assessed trainee improvement by having SPs score them during SP encounters before and after the workshop. Another shortcoming was the loss of follow-up of 12 of the original 43 interns who participated in the preworkshop survey. It is unclear whether the interns who chose not to or forgot to complete the postworkshop survey would have influenced the overall survey data differently.

This study lays the groundwork in demonstrating the efficacy of half-day workshop in educating internal medicine interns in EOL/BBN communication skills and improving confidence of interns in leading these conversations with patients. The half-day EOL-care/BBN workshop is designed to fit into the busy schedule of internal medicine interns more easily, which is an issue for full- or multiday workshops shown to be efficacious on the subject. A larger study utilizing stricter standardization of SPs and objective scoring of interns would be valuable in better assessing the efficacy of the half-day workshop in teaching EOL-care/BBN skills.

## Abbreviations Used

ACGME: Accreditation Counsel for Graduate Medical Education
BBN: breaking-bad-news
BWH: Brigham and Women's Hospital
EOL: end-of-life
PGY-1: postgraduate year-1
SP: standardized patient
